# Buffalo Academy of Medicine

**Published:** 1906-07

**Authors:** Franklin W. Barrows

**Affiliations:** Secretary


### Buffalo Academy of Medicine.

*Section of Medicine, May 8, 1906.*

Reported By FRANKLIN W. BARROWS, M. D., Secretary.

The regular meeting of the Section of Medicine was held in the Academy Rooms, Public Library Building, Tuesday Evening, May 8, 1906. The Section was called to order by the Chairman, Dr. A. W. Bayliss, at 8:55 p. m., after which the minutes of the previous meeting were read and approved.

The election of officers for the ensuing year resulted in the choice of Dr. T. J. Walsh, Secretary, and Dr. Franklin W. Barrows, Chairman.

PRESENTATION OF CASES

Dr. Grover W. Wende exhibited a case of Raynaud's Disease of three years duration, in a woman 34 years of age. The patient presented evidences of the disease in her fingers, toes, and face, showing the characteristic lesions of the three successive stages. At Dr. Van Peyma's request, Dr. Wende gave a history of the case with a concise exposition of the disease.

Dr. Albert J. Colton reported a case of tetany occurring in his practice, it being the second attack the patient had in a year. The patient is an adult. The contractures were somewhat atypical. He described the course of the disease and his treatment.

Dr. Eli H. Long, read a paper on

THE ADMINISTRATION OF IRON

reporting the clinical histories and data of a considerable number of cases of anemia, in which he had made systematic comparisons of results obtained with several varieties of iron peptonate and inorganic iron compounds, respectively. His experiments led him to conclude that the preparations of organic iron possess no advantage over the older forms of the pharmacopeia and that in some cases they produced very inferior results, as shown by blood counts and estimations of hemoglobin.

DISCUSSION

Dr. Delancey Rochester believed that Dr. Long's cases had merely borne out the conclusions reported by other observers—namely, that the tincture of the chloride is the best iron preparation for increasing hemoglobin, and that the carbonate, in the form of Vallet's mass or Blaud's pill, both of which should be freshly prepared, ranks next. He has discarded all other iron preparations in favor of these, and, sometimes, Basham's mixture.

Drs. Woodruff, Van Peyma, Bayliss, Dunham, Dwyer and Gardner continued the discussion.

DR. LONG, in conclusion, said that it was satisfactorily demonstrated that the inorganic preparations of iron are therapeutically superior to the great majority of newer forms of organic iron. The saccharated carbonate takes equal rank with Blaud's pill and the tincture of the chloride. He has never crowded the dose of Blaud's pill beyond three in one day.

DR. ALBERT T. LYTLE read a paper entitled

DROPSY

in which he described the physiology of the lymph circulation and reviewed the several varieties of dropsy with special reference to the attending changes in structure and functions of cells and tissues. The doctor cited a number of conditions in which dropsy, although playing an important role, is often overlooked or ignored.

DISCUSSION

DR. ROCHESTER, alluding to the mention of edema resulting from the employment of antitoxin, thought that a similar danger attended the use of other forms of serum medication, especially when horse serum is used. Allowance should be made, however, for the fact that in several reported cases of death from serum medication the patient was in extremis and serum was injected as a last resort. He had never seen actual evidences of edema after the giving of nucleinic acid, but on several occasions had observed cyanosis and rapid respiration which passed away in ten minutes or so. He had seen similar disturbances follow the use of antitubercle serum and Maragliano's serum. The cause in these cases is probably central.

DR. VAN PEYMA asked for an explanation of the production of pulmonary edema in cases of eclampsia.

DR. LYTLE, in conclusion, said that the edemas produced by the use of serums and nucleinic acid are similar to angioneurotic edema,—to the conditions existing in asthma, and edema of the larynx. In reply to Dr. Van Peyma he cited Welch's theory of the cause of edema of the lungs—namely, that it is due to a loss of equilibrium between the output of the right ventricle and that of the left ventricle. Such a loss of equilibrium may possibly occur in eclampsia, and result in pulmonary engorgement. Still another theory has been advanced, according to which any acid condition of the blood may result in pulmonary edema. Does such a condition exist in eclampsia?

The Section adjourned at 10:20. Attendance 23.

